# Warm acupuncture combined with precise meridian sinew needling for refractory peripheral facial paralysis: a propensity score-matched retrospective study on clinical efficacy and infrared thermal imaging

**DOI:** 10.3389/fmed.2026.1890419

**Published:** 2026-08-17

**Authors:** Yutong Jin, Yuxuan Zhang, Lihua Xuan

**Affiliations:** 1 Department of Acupuncture and Moxibustion, The First Affiliated Hospital of Zhejiang Chinese Medical University (Zhejiang Provincial Hospital of Chinese Medicine), Hangzhou, Zhejiang, China; 2 The First Clinical Medical College of Zhejiang Chinese Medical University, Hangzhou, Zhejiang, China

**Keywords:** clinical efficacy, infrared thermal imaging, precise meridian sinew needling, refractory peripheral facial paralysis, warm acupuncture

## Abstract

**Background:**

Refractory peripheral facial paralysis (RPFP) affects 15%–20% of patients with facial palsy, leading to poor recovery and reduced quality of life. Current treatments yield limited efficacy, and objective evidence for acupuncture-based therapies remains scarce.

**Methods:**

We addressed this gap by conducting a propensity score-matched retrospective study in 166 RPFP patients (H-B grade ≥ III, duration ≥ 3 months). Patients were divided into an observation group (warm acupuncture plus precise meridian sinew needling, *n* = 83) and a control group (conventional acupuncture, *n* = 83). Outcomes included clinical efficacy, TCM syndrome scores, facial nerve function (H-B grade, Portmann score), facial disability index (FDI), safety, and infrared thermal imaging parameters (bilateral ΔT, ROI ΔT, LBP histogram distance).

**Results:**

The observation group showed significantly higher cure rate (59.04% vs. 43.37%, *P* = 0.044) and total effective rate (93.98% vs. 80.72%, *P* = 0.010). After treatment, TCM syndrome scores, H-B grade, bilateral ΔT (0.26 ± 0.05 vs. 0.32 ± 0.08°C), ROI ΔT (0.28 ± 0.07 vs. 0.35 ± 0.08°C), and LBP distance (0.39 ± 0.09 vs. 0.48 ± 0.10) were all significantly lower in the observation group (all *P* < 0.001); Portmann score and FDI subscales were significantly higher (all *P* < 0.001). The observed adverse-event proportions did not differ statistically; this finding does not establish safety equivalence.

**Conclusion:**

In this propensity score-matched retrospective study, warm acupuncture combined with precise meridian sinew needling was associated with better short-term clinical, functional, and facial thermal-symmetry outcomes than conventional acupuncture. Infrared thermography quantified surface-temperature asymmetry but did not directly measure blood flow. Prospective multicentre studies with longer follow-up are needed to confirm these findings.

## 1 Introduction

Peripheral facial paralysis (PFP) is a common neurological disorder characterized by dysfunction of the facial nerve (cranial nerve VII), with a global annual incidence rate of approximately 20–30 cases per 100,000 population (1, 2). The disease manifests as unilateral facial muscle weakness or paralysis, including inability to raise the eyebrow, incomplete eye closure, and deviation of the mouth angle, which profoundly impairs patients’ facial expression function, daily activities, social interactions, and psychological wellbeing (3, 4). Although approximately 70%–85% of patients with PFP achieve satisfactory functional recovery with standard medical management, a substantial proportion—estimated at 15%–20%—progress to refractory peripheral facial paralysis (RPFP) despite receiving conventional treatment (5, 6). Moreover, up to 40% of patients may experience permanent sequelae such as synkinesis, muscle contracture, and crocodile tears syndrome (2). RPFP is generally defined by a disease duration exceeding three months with persistent House-Brackmann (H-B) grade of III or higher, representing a severe clinical entity associated with extensive axonal degeneration, impaired nerve regeneration, and compromised facial microcirculation (7, 8). These patients often endure prolonged recovery trajectories, unfavorable prognoses, and significant residual facial disability, underscoring the urgent need for more effective therapeutic strategies for this challenging condition (9, 10).

Conventional Western medical management for RPFP primarily includes neurotrophic agents (e.g., mecobalamin, vitamin B complex), vasoactive drugs (e.g., corticosteroids, prostaglandins), physical therapy (including facial muscle rehabilitation and electrical stimulation), and, in selected refractory cases, surgical decompression (11, 12). However, the efficacy of these interventions remains unsatisfactory for patients with established nerve pathology beyond the acute phase (13). A systematic review and meta-analysis of 14 randomized controlled trials involving electrical stimulation and proprioceptive neuromuscular facilitation for PFP concluded that while such modalities show modest benefits, significant heterogeneity in treatment protocols and outcome measures precludes definitive conclusions about their optimal application in refractory cases (2). Furthermore, a recent overview of systematic reviews including 17 SRs/MAs on acupuncture for PFP revealed that while acupuncture can improve clinical efficacy and reduce recovery time, most included studies suffered from low or very low methodological quality according to AMSTAR2 and GRADE assessments (14, 15). The long-standing dysfunction of the facial nerve and persistent ischemic changes in RPFP often render isolated pharmacotherapy insufficient to reverse established neuropathological damage. In contrast, acupuncture, as a cornerstone of Traditional Chinese Medicine (TCM), has been widely recognized as a safe and effective treatment for various neurological disorders (6). Systematic reviews have demonstrated that acupuncture significantly improves clinical effective rates (RR = 1.11, 95% CI [1.06, 1.16]), reduces H-B grade scores (MD = −0.56, 95% CI [−0.92, −0.20]), and shortens recovery time (MD = −10.71 days, 95% CI [−16.33, −5.09]) when combined with conventional therapy for PFP (16). However, the vast majority of existing acupuncture research has focused on acute and subacute PFP (disease duration < 3 months). Robust evidence specifically targeting the RPFP population—characterized by chronicity, nerve scarring, and microcirculatory impairment—remains conspicuously limited (17). An umbrella review of non-pharmacological interventions for facial paralysis noted that while acupuncture may enhance recovery rates, evidence is constrained by high heterogeneity and methodological limitations, particularly in studies focusing on chronic or refractory cases (18). Collectively, the above-mentioned findings indicate that current treatments for RPFP are suboptimal and that rigorously designed studies evaluating novel acupuncture-based combination therapies for this difficult-to-treat population are urgently needed.

Traditional Chinese Medicine theory posits that refractory peripheral facial paralysis fundamentally stems from a pattern of “Qi deficiency with blood stasis” and “deficiency with pathogen retention” (19). The extended disease course depletes vital energy, impairs nourishment of facial meridians, and leads to blood stagnation within the collateral channels (20). According to *Ling Shu⋅Meridian Sinews*, diseases of the meridian sinews manifest as rigidity and spasm with cold patterns or flaccidity with heat patterns (21). The facial region is traversed by the Yangming meridian sinews, including the Stomach Meridian of Foot-Yangming and the Large Intestine Meridian of Hand-Yangming, and dysfunctions in these sinews constitute the primary pathological basis of facial paralysis (22). Treatment of RPFP should therefore adhere to the fundamental principles of “supplementing Qi and nourishing blood, warming meridians and unblocking collaterals, and resolving stasis to relax the sinews.” Warm acupuncture, a specialized acupuncture technique, combines traditional needling with moxibustion by placing moxa on the needle handle and igniting it to conduct heat deep into the acupoint (23). This approach concurrently provides mechanical stimulation through needle insertion and thermal effects through moxa combustion, thereby warming meridians, dispelling cold, resolving stasis, and promoting Qi and blood circulation. Clinical studies have shown that warm acupuncture improves total effective rates and facial nerve function scores in PFP patients (13). A recent prospective trial of 84 elderly patients with refractory facial paralysis demonstrated that warm acupuncture combined with low-frequency pulsed electrical stimulation significantly improved Sunnybrook facial nerve assessment scores and FDIP/FDIS scores compared with warm acupuncture alone, indicating synergistic benefits of thermal and electro-stimulation modalities (24). Additionally, thermosensitive moxibustion at the Yifeng acupoint has been shown to enhance total effective rates and alleviate anxiety and depression in patients with intractable facial paralysis (25, 26). Precise meridian sinew needling, grounded in the meridian sinew theory, employs multi-site, multi-directional needling techniques such as fan-shaped penetration and palisade-like array needling to extensively activate motor endplate zones of facial expression muscles, enhancing local neuromuscular feedback (27, 28). A recent meta-analysis of 19 randomized controlled trials involving 1,436 patients reported that Jingjin (meridian sinew) acupuncture therapy significantly improved overall effectiveness rate (OR = 3.93, 95% CI [2.78, 5.56]) and cure rate (RR = 1.69, 95% CI [1.51, 1.90]) compared with conventional acupuncture, along with superior FDIP, FDIS, Portmann, and facial nerve function scores (29). However, the authors noted that the reliability of these findings is constrained by the limited number of high-quality studies, and no trials specifically addressing RPFP with combined warm acupuncture and meridian sinew needling have been published (29). Moreover, despite the established theoretical rationale, no study has integrated infrared thermal imaging (ITI) as an objective outcome measure to evaluate treatment-induced changes in facial microcirculation, which represent a core pathophysiological feature of RPFP (30). ITI can non-invasively quantify facial temperature symmetry by calculating parameters such as bilateral temperature difference (ΔT), region of interest (ROI) ΔT, and Local Binary Pattern (LBP) histogram distance, and these parameters correlate positively with the severity of facial paralysis (31–34). A study by Liu et al. demonstrated that the LBP histogram distance in facial infrared thermography achieved a mean sensitivity of 0.86 and specificity of 0.89 for assessing the severity of facial paralysis, with the asymmetry degree positively correlated with clinical severity scores (mean correlation = 0.657) (35). Another study reported that infrared thermal imaging can effectively diagnose early-onset Bell‘s palsy with an AUC of 0.818 for ΔTroi in the infraorbital region (36). Furthermore, a randomized controlled trial evaluating meridian sinew needling for refractory facial paralysis using ITI confirmed that this method significantly reduced the facial temperature difference between the healthy and affected sides and promoted microcirculation in the affected side (37). Consequently, the combination of warm acupuncture and precise meridian sinew needling, evaluated with objective ITI endpoints, may fill a critical gap in current RPFP research.

Therefore, the present study was designed to evaluate the clinical efficacy of warm acupuncture combined with precise meridian sinew needling in patients with refractory peripheral facial paralysis (disease duration ≥ 3 months, H-B grade ≥ III, TCM pattern of Qi deficiency and blood stasis). Using a propensity score-matched retrospective design, we compared this combined therapy to conventional acupuncture, with outcome measures including clinical efficacy (cure rate and total effective rate), TCM syndrome scores, facial nerve function (H-B grade and Portmann scores), Facial Disability Index (FDI, including FDIP and FDIS subscales), treatment safety, and the key objective endpoint of infrared thermal imaging parameters (bilateral ΔT, ROI ΔT, LBP histogram distance). This study aims to provide high-quality evidence for an optimized acupuncture-based regimen for RPFP and to offer theoretical guidance for clinical decision-making in the management of this challenging patient population.

## 2 Materials and methods

This retrospective study was conducted in accordance with the Declaration of Helsinki and was approved by the Institutional Review Board of The First Affiliated Hospital of Zhejiang Chinese Medical University (Approval No. 2026-KLS-301-02, approved on 2 January 2026). Due to the retrospective nature of the analysis, the requirement for informed consent was waived. The study is reported following the STROBE (Strengthening the Reporting of Observational Studies in Epidemiology) statement for observational research.

### 2.1 Diagnostic criteria

The diagnosis of peripheral facial paralysis was established according to the criteria outlined in Adams and Victor’s Principles of Neurology (7) and the clinical practice guideline for Bell’s palsy (1). Patients were diagnosed with peripheral facial paralysis if they presented with unilateral complete or partial paralysis of facial expression muscles, manifested by disappearance or flattening of forehead wrinkles, shallowing of the nasolabial fold, incomplete eyelid closure, and deviation of the mouth angle to the healthy side, with or without ipsilateral loss of taste on the anterior two-thirds of the tongue. Central lesions were excluded by neurological examination and, when indicated, neuroimaging (7).

The Traditional Chinese Medicine (TCM) pattern of Qi deficiency and blood stasis was diagnosed according to the criteria described in the literature (19, 20). The primary symptoms included: long-standing facial deviation and eye–mouth distortion (≥3 months), facial numbness or rigidity, incomplete eyelid closure, drooling, and facial muscle atrophy or spasm on the affected side. Secondary symptoms included: sallow or dark facial complexion, fatigue, shortness of breath, reluctance to speak, dizziness, blurred vision, limb numbness, and poor appetite. Tongue examination: pale or dark tongue with or without ecchymosis or petechiae. Pulse examination: thready, rough, or deep and weak pulse. Patients meeting the primary symptom criteria plus at least two secondary symptoms, along with the tongue and pulse findings, were classified as having the Qi deficiency and blood stasis pattern (10). Because eligibility was restricted to this pattern, the analyzed cohort cannot estimate its prevalence among all patients with RPFP. Patients with other patterns, including phlegm with blood stasis, were outside the study population, and the findings should not be generalized to those groups.

### 2.2 Inclusion and exclusion criteria

Inclusion criteria: (1) met the above-mentioned Western medical and TCM diagnostic criteria for peripheral facial paralysis; (2) disease duration ≥ 3 months (refractory peripheral facial paralysis, RPFP); (3) TCM pattern confirmed as Qi deficiency and blood stasis; (4) House–Brackmann (H-B) grade ≥ III (8); (5) age between 18 and 75 years; (6) able to tolerate acupuncture treatment. The 18–75-year criterion defined the adult study population. The TCM pattern was determined using the prespecified symptom, tongue, and pulse criteria rather than age alone, and age was included in propensity-score matching. However, the study was not designed or powered to assess age-specific treatment effects; residual age-related confounding and limited generalizability to adults older than 75 years should therefore be considered.

Exclusion criteria: (1) central facial paralysis (confirmed by neuroimaging or neurological examination); (2) pregnancy or lactation; (3) facial skin lesions, wounds, or local infections that would contraindicate needling; (4) concurrent participation in other treatment interventions (e.g., other acupuncture, electrical stimulation, or investigational drugs) within the past 2 weeks.

### 2.3 Study participants and propensity score matching

This was a retrospective analysis of patients with refractory peripheral facial paralysis admitted to our hospital between January 2023 and December 2025. To minimize confounding bias, propensity score matching (PSM) was performed in a 1:1 ratio using the following matching variables: age, sex, disease duration, and pre-treatment H-B grade. A logistic regression model was used to calculate the propensity score for each patient, and nearest-neighbor matching with a caliper width of 0.2 times the standard deviation of the logit-transformed propensity score was applied. Treatment allocation was not randomized and reflected the treatment documented in routine clinical records; therefore, confounding by indication cannot be excluded.

After matching, a total of 166 patients were successfully matched, with 83 patients in each group. Observation group (warm acupuncture + precise meridian sinew needling): 40 males and 43 females; age range 18–72 years (mean 44.39 ± 6.24 years); body mass index (BMI) range 18–28 kg/m^2^ (mean 22.73 ± 2.93 kg/m^2^); disease duration range 3–11 months (mean 7.12 ± 1.93 months); H-B grade distribution: grade III, *n* = 14; grade IV, *n* = 28; grade V, *n* = 30; grade VI, *n* = 11. Control group (conventional acupuncture): 44 males and 39 females; age range 20–75 years (mean 45.32 ± 6.11 years); BMI range 19–28 kg/m^2^ (mean 22.97 ± 2.90 kg/m^2^); disease duration range 4–12 months (mean 7.64 ± 1.87 months); H-B grade distribution: grade III, *n* = 15; grade IV, *n* = 31; grade V, *n* = 29; grade VI, *n* = 8. There were no statistically significant differences in baseline characteristics between the two groups (all *P* > 0.05), indicating successful matching.

Using the reported post-matching aggregate data, the absolute standardized mean differences were 0.151 for age, 0.097 for sex, 0.274 for disease duration, and 0.106 for baseline H-B grade calculated from the reported grade counts. These diagnostics indicate residual imbalance in age and disease duration. Pre-matching SMDs, a Love plot, common-support information, missing-data handling, and unmatched-patient counts require the original patient-level dataset and were not reconstructed.

### 2.4 Treatment protocols

All acupuncture procedures were performed by licensed acupuncturists with at least 5 years of clinical experience in treating facial paralysis. Each patient received treatment once daily, six times per week (Sunday off), for a total of 4 weeks (24 sessions). According to the treatment protocol documented for this retrospective cohort, facial acupoints and facial meridian-sinew needling sites were prescribed on the affected side, whereas Hegu (LI4) was prescribed contralaterally (on the healthy side). Because individual treatment records did not consistently document point laterality for every session, adherence to this laterality protocol could not be verified at the session level.

#### 2.4.1 Control group: conventional acupuncture

Conventional acupuncture was administered based on standard protocols (6, 16). The following main acupoints were selected: Yangbai (GB14), Sibai (ST2), Dicang (ST4), Jiache (ST6), Hegu (LI4), and Taichong (LR3). Supplementary acupoints were added according to syndrome differentiation [e.g., Zusanli (ST36) for Qi deficiency, Sanyinjiao (SP6) for blood stasis]. After routine sterilization, disposable stainless steel acupuncture needles (0.25 mm × 40 mm for facial points, 0.30 mm × 50 mm for limb points) were inserted perpendicularly or obliquely to a depth of 0.5–1.5 cun depending on the acupoint location. The even-needling technique was applied until deqi (needling sensation) was achieved. Needles were retained for 30 min. Treatment was given once daily, six times per week for 4 weeks (6).

#### 2.4.2 Observation group: warm acupuncture combined with precise meridian sinew needling

The observation group received precise meridian sinew needling combined with warm acupuncture at selected acupoints, based on meridian sinew theory and previous studies (29, 37). Precise meridian sinew needling consisted of three specific techniques: The observation group received this combined protocol instead of the conventional-acupuncture protocol; consequently, the study estimates the effect of the complete treatment package and cannot isolate the contribution of any single component, needle number, treatment intensity, or therapist contact. Accordingly, a factorial or multi-arm dismantling trial is required to evaluate precise meridian sinew needling and warm acupuncture separately.

(1) Yangbai four-directional penetration needling: Using a 0.25 mm × 40 mm needle inserted at Yangbai (GB14) at a 15° angle along the skin, the needle was directed toward Shangxing (GV23), Touwei (ST8), Cuanzhu (BL2), and Sizhukong (TE23) in four separate penetrations. The needling depth was 0.5–1 cun subcutaneously. Even reinforcing–reducing manipulation by twisting was applied until deqi was obtained. This technique targets the motor endplate zones of the frontal belly of the occipitofrontalis muscle (29).

(2) Dicang–Jiache palisade needling: Along the line from Dicang (ST4) to Jiache (ST6), 6–8 needles (0.25 mm × 25 mm) were inserted at intervals of approximately 0.5 cun (1.5 cm) at a 15° angle along the skin, with the needle tip pointing toward the angle of the mouth. Needle depth was 0.5 cun subcutaneously. No vigorous twisting was performed to avoid excessive stimulation. This palisade-like array activates the orbicularis oris and buccinator muscles (37).

(3) Taiyang (EX-HN5) to Dicang penetration needling: A 0.30 mm × 50 mm needle was inserted at Taiyang (EX-HN5) at a 30° angle obliquely downward toward Dicang (ST4), to a depth of 1–1.5 cun. Even reinforcing–reducing manipulation by twisting was applied. This technique connects the innervation territories of the zygomatic and buccal branches of the facial nerve (29).

Warm acupuncture was applied at Zusanli (ST36), Guanyuan (CV4), and Mingmen (GV4) (24, 25). After routine needling and obtaining deqi, a 2 cm moxa stick (manufactured by Nanyang Hanyi Moxa Co., Ltd., China; 4:1 wormwood-to-moxa ratio) was placed on the needle handle and ignited at the distal end. Each acupoint received two moxa cones (about 15–20 min each) until local skin flushing was observed. The moxa ash was carefully removed after each cone. This combined technique provides both needle stimulation and thermal effect to warm meridians, dispel cold, resolve stasis, and promote Qi and blood circulation (24, 25).

All treatments in the observation group were administered once daily, six times per week for 4 weeks, with the same frequency and total duration as the control group.

### 2.5 Outcome measures

All outcome assessments were performed by trained evaluators who were blinded to group allocation (assessor-blinded design). Outcomes were measured at baseline (before treatment) and at the end of the 4-week treatment period. No outcome data were available beyond the end of treatment; thus, this study cannot determine the durability of benefit or relapse after treatment.

#### 2.5.1 Primary outcome: clinical efficacy

Clinical efficacy was evaluated using the criteria adapted from the literature (11, 12, 29): Cure: Facial expression muscle function returned to normal, with H-B grade I (8). Significant effect: Facial expression muscle function markedly improved, with H-B grade improvement ≥ 2 levels. Effective: Facial expression muscle function somewhat improved, with H-B grade improvement by 1 level. Ineffective: No significant improvement in symptoms after treatment. Total effective rate = (cure + significant effect + effective)/total number of patients × 100%. Cure rate = (cure)/total number of patients × 100%. Because the total effective rate is defined largely by change in H-B grade, it overlaps with the separately reported H-B outcome and should not be interpreted as an independent confirmation of efficacy.

#### 2.5.2 Secondary outcomes

(1) Traditional Chinese Medicine (TCM) syndrome scores: Four main symptoms—(a) deviation of mouth and eye, (b) facial numbness, (c) sallow complexion, and (d) pale tongue with whitish coating—were each rated on a scale of 0, 2, 4, and 6 representing none, mild, moderate, and severe, respectively (19, 20). Lower scores indicate less severe TCM symptoms.

(2) Facial nerve function scores: House–Brackmann (H-B) grading scale: Grades I to VI were converted to scores of 1–6, with lower scores indicating better facial nerve function (8). Portmann score: This scale comprises resting state (2 points) and voluntary movement (18 points, including 6 items: forehead wrinkling, eye closure, mouth corner elevation, showing teeth, whistling/wrinkling nose, and lower lip depression), with a maximum total of 20 points. Higher scores indicate better facial function (13).

(3) Infrared thermal imaging (ITI) parameters: ITI was performed using an ATIR-M301B infrared thermal imager (Chongqing Weilian, China; temperature resolution ≤ 0.05 °C, operating waveband 8–14 μm) in a temperature-controlled room (20 °C–25 °C), constant humidity, draft-free, and without direct sunlight, according to standardized protocols (4, 31, 36). Patients rested in a seated position for 15–20 min to achieve thermal equilibrium, removed facial accessories, and kept their eyes level looking forward with relaxed facial muscles. Three thermal images were captured: frontal view, 45° left oblique, and 45° right oblique. The following parameters were calculated: Bilateral temperature difference (ΔT): Absolute temperature difference between the affected and healthy sides. Healthy individuals typically have ΔT < 0.2 °C; mild-to-moderate facial paralysis shows regional ΔT 0.2 °C–0.5 °C; severe paralysis shows ΔT > 0.5 °C (36). Region of interest (ROI) ΔT: Six symmetric facial ROIs (frontal region, infraorbital region, perioral region, etc.) were selected, and the mean temperature difference between the affected and healthy sides was calculated (4, 31). LBP histogram distance: a local binary pattern (LBP) texture descriptor was extracted from the thermal images to quantify facial temperature distribution symmetry. Smaller LBP histogram distance indicates more symmetrical temperature distribution (35). The stated thermal resolution is not equivalent to absolute accuracy, repeatability, or a clinically meaningful change. Camera calibration, emissivity, acquisition distance and angle, exact ROI coordinates, image-processing procedures, and reliability estimates should be reported from the original imaging protocol when available. In RPFP, which represents a chronic or sequela-stage condition, facial thermal asymmetry may be smaller than in the acute stage. This may reduce sensitivity to treatment-related change and create a floor effect; therefore, ITI was interpreted as an exploratory adjunct to, rather than a replacement for, clinical and patient-reported outcomes.

(4) Prognostic effect: Facial Disability Index (FDI): The FDI questionnaire includes two subscales: FDIP (physical function, 10 items) and FDIS (social/wellbeing function, 5 items). Each subscale score was converted to a 0–100 scale, with higher scores indicating better physical and social function (2, 8).

(5) Treatment safety: Adverse events (e.g., needling - induced nausea, hematoma, infection, burn from moxibustion) were recorded and compared between groups. All events were graded for severity. Documented preventive measures included routine skin disinfection, use of disposable sterile needles, and careful removal of moxa ash after each cone. Recorded adverse events were mild and resolved spontaneously or after symptomatic management. Because the retrospective records did not contain a standardized session-level safety checklist, additional prevention and management steps could not be verified.

### 2.6 Statistical analysis

Statistical analyses were performed using SPSS version 25.0 (IBM Corp., Armonk, NY, USA). Continuous variables are presented as mean ± standard deviation (SD), and categorical variables as frequencies and percentages (*n*, %). Between-group comparisons for continuous variables were performed using independent-samples *t*-tests after testing for normality (Shapiro–Wilk test) and homogeneity of variances (Levene‘s test). For non-normally distributed data, the Mann–Whitney U test was used as a sensitivity analysis. Categorical variables were compared using the chi-square (χ^2^) test or Fisher’s exact test, as appropriate. All tests were two-tailed, and a *P*-value < 0.05 was considered statistically significant. No interim analyses were performed, and no adjustment for multiple comparisons was made because the secondary outcomes were pre-specified and exploratory. The total effective rate was treated as the primary outcome; all other outcomes were secondary and exploratory. The effect estimates below were calculated from the reported aggregate summaries and use unpaired large-sample approximations. They do not replace a participant-level analysis that accounts for matched pairs, baseline values, ordinal outcomes, and multiplicity. The available manuscript reports within-group pre-treatment versus post-treatment comparisons, but the original participant-level dataset and archived statistical output required to confirm whether paired-samples *t*-tests or non-parametric paired tests were applied were unavailable for this revision. These within-group *P*-values should therefore be interpreted cautiously; the principal comparative inference is based on the reported between-group analyses.

## 3 Results

### 3.1 Clinical efficacy

After the 4-week treatment period, the clinical efficacy was evaluated and compared between the two groups (Table 1). In the observation group (warm acupuncture combined with precise meridian sinew needling), 49 out of 83 patients (59.04%) achieved cure, 17 patients (20.48%) showed significant effect, and 12 patients (14.46%) showed effective response, while only five patients (6.02%) were classified as ineffective. Consequently, the total effective rate in the observation group was 93.98% (78/83). In the control group (conventional acupuncture), 36 patients (43.37%) achieved cure, 18 patients (21.69%) showed significant effect, and 13 patients (15.66%) showed effective response, whereas 16 patients (19.28%) were ineffective, yielding a total effective rate of 80.72% (67/83). The observation group demonstrated a significantly higher cure rate (59.04% vs. 43.37%, χ^2^ = 4.075, *P* = 0.044) and a significantly higher total effective rate (93.98% vs. 80.72%, χ^2^ = 6.596, *P* = 0.010) compared with the control group. These results indicate that the combination therapy is superior to conventional acupuncture in improving overall clinical outcomes and achieving complete recovery in patients with refractory peripheral facial paralysis (11, 29). From the aggregate counts, the cure-rate risk ratio was 1.36 (95% CI 1.00–1.85) and the risk difference was 15.7 percentage points (95% CI 0.6 to 30.7). The total-effective-rate risk ratio was 1.16 (95% CI 1.03–1.31) and the risk difference was 13.3 percentage points (95% CI 3.3 to 23.2).

**TABLE 1:** Comparison of clinical effective rates [*n* (%)].

Group	*n*	Recovery	Significant effect	Effective	Invalid	Overall efficiency	Recovery rate
Observation group	83	49 (59.04)	17 (20.48)	12 (14.46)	5 (6.02)	78 (93.98)	49 (59.04)
Control group	83	36 (43.37)	18 (21.69)	13 (15.66)	16 (19.28)	67 (80.72)	36 (43.37)
*χ^2^*	–	–	–	–	–	6.596	4.075
*P*	–	–	–	–	–	0.010	0.044

### 3.2 Traditional Chinese Medicine syndrome scores

As presented in Table 2, the two groups were comparable at baseline across all four TCM syndrome items (all *P >* 0.05). After treatment, both groups showed significant improvements in each item compared with their respective baseline values (all *P <* 0.05). More importantly, the post-treatment scores in the observation group were significantly lower than those in the control group for all four symptoms. Specifically, for deviation of mouth and eye, the observation group scored 1.72 ± 0.33 versus 1.97 ± 0.39 in the control group (*t* = 4.458, *P <* 0.001). For facial numbness, the scores were 1.83 ± 0.38 and 2.17 ± 0.43, respectively (*t* = 5.398, *P <* 0.001). For sallow complexion, the observation group scored 1.90 ± 0.42 compared with 2.28 ± 0.47 in the control group (*t* = 5.492, *P <* 0.001). For pale tongue with whitish coating, the scores were 1.98 ± 0.43 versus 2.38 ± 0.50 (*t* = 5.526, *P <* 0.001). These findings demonstrate that warm acupuncture combined with precise meridian sinew needling is more effective than conventional acupuncture in alleviating the TCM syndrome manifestations of Qi deficiency and blood stasis in RPFP patients (19, 20). Aggregate-data mean differences (observation minus control) were −0.25 (95% CI −0.36 to −0.14) for deviation of the mouth and eye, −0.34 (95% CI −0.46 to −0.22) for facial numbness, −0.38 (95% CI −0.52 to −0.24) for sallow complexion, and −0.40 (95% CI −0.54 to −0.26) for pale tongue with whitish coating.

**TABLE 2:** Comparison of Traditional Chinese Medicine (TCM) syndrome scores between the two groups (mean ± SD).

Group	*n*	Oblique mouth and eyes		Facial numbness		Pale complexion		Pale tongue with whitish coating	
		Before treatment	After treatment	Before treatment	After treatment	Before treatment	After treatment	Before treatment	After treatment
Observation group	83	5.13 ± 0.62	1.72 ± 0.33*	4.98 ± 0.78	1.83 ± 0.38*	4.89 ± 0.68	1.90 ± 0.42*	4.71 ± 0.83	1.98 ± 0.43*
Control group	83	5.03 ± 0.61	1.97 ± 0.39*	5.06 ± 0.62	2.17 ± 0.43*	4.72 ± 0.70	2.28 ± 0.47*	4.80 ± 0.81	2.38 ± 0.50*
*t*		1.047	4.458	0.731	5.398	1.587	5.492	0.707	5.526
*P*		0.296	0.000	0.466	0.000	0.114	0.000	0.481	0.000

Compared with the patients in this group before treatment.

*P < 0.05

### 3.3 Facial nerve function scores

The facial nerve function was assessed using the H-B grading scale (lower score indicates better function) and the Portmann scale (higher score indicates better function). At baseline, no significant differences existed between the two groups in either H-B score or Portmann score (both *P >* 0.05). After treatment, both groups exhibited significant improvement from baseline (all *P <* 0.05). As shown in Table 3, the observation group achieved a significantly lower H-B score (1.69 ± 0.40) compared with the control group (2.02 ± 0.48), with a mean difference of −0.33 (95% CI not calculated but *t* = 4.812, *P <* 0.001). In contrast, the Portmann score was significantly higher in the observation group (17.02 ± 2.16) than in the control group (15.30 ± 2.41), with a mean difference of 1.72 points (*t* = 4.842, *P <* 0.001). These results indicate that the combination therapy produces superior restoration of facial nerve function, including both resting symmetry and voluntary movement, compared with conventional acupuncture alone (8, 13). Aggregate-data mean differences were −0.33 (95% CI −0.46 to −0.20) for H-B score and 1.72 (95% CI 1.02 to 2.42) for Portmann score. These unpaired estimates require confirmation using the matched participant-level data. The baseline H-B means in Table 3 should also be reconciled with the grade counts reported in section “2.3 Study participants and propensity score matching.”

**TABLE 3:** Comparison of facial nerve function scores (score, $\bar{\chi }$ ± s).

Group	*n*	H-B grading score		Portmann score	
		Before treatment	After treatment	Before treatment	After treatment
Observation group	83	4.36 ± 0.67	1.69 ± 0.40*	9.88 ± 2.65	17.02 ± 2.16*
Control group	83	4.22 ± 0.65	2.02 ± 0.48*	9.97 ± 2.43	15.30 ± 2.41*
*t*		1.367	4.812	0.228	4.842
*P*		0.174	0.000	0.820	0.000

Compared with the patients in this group before treatment.

*P < 0.05.

### 3.4 Infrared thermal imaging parameters

Infrared thermal imaging provided objective quantification of facial surface-temperature symmetry; it did not directly quantify microcirculation or perfusion. The three parameters measured were bilateral temperature difference (ΔT, in °C), region of interest (ROI) ΔT (in °C), and LBP histogram distance (dimensionless, smaller values indicate more symmetric temperature distribution). At baseline, no significant differences were observed between the two groups for any of the three parameters (all *P* > 0.05). After treatment, both groups showed significant reductions in all parameters compared with baseline (all *P* < 0.05). Critically, the observation group demonstrated significantly lower values than the control group for all three post-treatment parameters (Table 4). Specifically, bilateral ΔT decreased to 0.26 °C ± 0.05 °C in the observation group versus 0.32 °C ± 0.08 °C in the control group (*t* = 5.794, *P* < 0.001). ROI ΔT decreased to 0.28 °C ± 0.07 °C in the observation group compared with 0.35 °C ± 0.08 °C in the control group (*t* = 5.999, *P* < 0.001). The LBP histogram distance was reduced to 0.39 ± 0.09 in the observation group versus 0.48 ± 0.10 in the control group (*t* = 6.095, *P* < 0.001). These objective thermal imaging findings confirm that warm acupuncture combined with precise meridian sinew needling is more effective than conventional acupuncture in reducing the inter-facial temperature difference, normalizing regional thermal asymmetry, and improving the overall symmetry of facial temperature distribution. Such improvements are consistent with enhanced local blood circulation and reduced vasomotor dysfunction in the affected facial region, as previously described in ITI studies of facial paralysis (4, 36). Aggregate-data mean differences were −0.06 °C (95% CI −0.08 to −0.04) for bilateral ΔT, −0.07 °C (95% CI −0.09 to −0.05) for ROI ΔT, and −0.09 (95% CI −0.12 to −0.06) for LBP histogram distance. The 0.06 °C bilateral difference is close to the stated device resolution and should not be interpreted as direct evidence of increased perfusion.

**TABLE 4:** Comparison of infrared thermal imaging index levels ($\bar{\chi }$ ± s).

Group	*n*	Bilateral ΔT (C°)		ROI ΔT (C°)		LBP histogram distance	
		Before treatment	After treatment	Before treatment	After treatment	Before treatment	After treatment
Observation group	83	0.49 ± 0.10	0.26 ± 0.05*	0.51 ± 0.12	0.28 ± 0.07*	0.69 ± 0.16	0.39 ± 0.09*
Control group	83	0.47 ± 0.09	0.32 ± 0.08*	0.48 ± 0.11	0.35 ± 0.08*	0.66 ± 0.15	0.48 ± 0.10*
*t*		1.354	5.794	1.679	5.999	1.246	6.095
*P*		0.177	0.000	0.095	0.000	0.214	0.000

Compared with the patients in this group before treatment.

*P < 0.05.

### 3.5 Prognostic effect scores (Facial Disability Index)

The Facial Disability Index (FDI) comprises two subscales: FDIP (physical function) and FDIS (social/wellbeing function), both converted to a 0–100 scale with higher scores indicating better quality of life. At baseline, the two groups had comparable FDIP and FDIS scores (both *P >* 0.05). After treatment, both groups showed significant improvements from baseline (all *P <* 0.05). As shown in Table 5, the observation group achieved significantly higher post-treatment scores than the control group for both subscales. The FDIP score increased to 83.92 ± 8.10 in the observation group versus 78.24 ± 7.98 in the control group (*t* = 4.551, *P <* 0.001). Similarly, the FDIS score increased to 85.22 ± 8.16 in the observation group compared with 77.93 ± 8.01 in the control group (*t* = 5.808, *P <* 0.001). These results indicate that the combination therapy not only improves facial motor function but also translates into better physical performance in daily activities (e.g., eating, drinking, eye closure) and enhanced social and emotional wellbeing (e.g., social interaction, work performance, mood) compared with conventional acupuncture (2, 8). Aggregate-data mean differences were 5.68 points (95% CI 3.23 to 8.13) for FDIP and 7.29 points (95% CI 4.83 to 9.75) for FDIS; clinical importance was not prespecified.

**TABLE 5:** Comparison of prognostic effect scores (score, $\bar{\chi }$ ± s).

Group	*n*	FDIP score		FDIS score	
		Before treatment	After treatment	Before treatment	After treatment
Observation group	83	56.36 ± 5.69	83.92 ± 8.10*	61.02 ± 5.93	85.22 ± 8.16*
Control group	83	57.52 ± 5.74	78.24 ± 7.98*	60.17 ± 6.07	77.93 ± 8.01*
*t*	–	1.308	4.551	0.913	5.808
*P*	–	0.193	0.000	0.363	0.000

Compared with the patients in this group before treatment.

*P < 0.05.

### 3.6 Treatment safety

Adverse events recorded during the treatment period included needling-induced nausea, hematoma at needle insertion sites, local infection, and moxibustion-related burns. As shown in Table 6, in the observation group, needling-induced nausea occurred in two patients (2.41%), hematoma in two patients (2.41%), local infection in two patients (2.41%), and burn in two patients (2.41%), resulting in an overall adverse event rate of 9.64% (8/83). In the control group, needling-induced nausea occurred in two patients (2.41%), hematoma in two patients (2.41%), local infection in one patient (1.20%), and burn in one patient (1.20%), with an overall adverse event rate of 7.23% (6/83). The difference in overall adverse event rates between the two groups was not statistically significant (χ^2^ = 0.312, *P* = 0.576). All adverse events were mild in severity and resolved spontaneously or after simple symptomatic management without sequelae. No serious adverse events (e.g., severe infection, nerve damage requiring hospitalization, or life-threatening conditions) were observed in either group. The aggregate-data risk ratio for experiencing a recorded adverse event was 1.33 (95% CI 0.48–3.67), and the risk difference was 2.4 percentage points (95% CI −6.0 to 10.9). The wide confidence intervals do not establish equivalent safety. The control-group burn must be checked against the source records because the stated control protocol did not include moxibustion.

**TABLE 6:** Comparison of treatment safety [*n* (%)].

Group	*n*	Nausea caused acupuncture	Hematoma	Infection	Burn	Overall incidence rate
Observation group	83	2 (2.41)	2 (2.41)	2 (2.41)	2 (2.41)	8 (9.64)
Control group	83	2 (2.41)	2 (2.41)	1 (1.20)	1 (1.20)	6 (7.23)
*χ^2^*	–	–	–	–	–	0.312
*P*	–	–	–	–	–	0.576

## 4 Discussion

### 4.1 Overview of the present study

In this propensity score-matched retrospective study of 166 patients with refractory peripheral facial paralysis (RPFP), we found that a 4-week course of warm acupuncture combined with precise meridian sinew needling produced significantly better clinical outcomes than conventional acupuncture. Specifically, the combination therapy yielded higher cure and total effective rates, greater improvements in TCM syndrome scores, superior facial nerve function (lower H-B grade and higher Portmann score), and better quality of life as measured by the Facial Disability Index (FDIP and FDIS). More importantly, using infrared thermal imaging as an objective endpoint, we demonstrated that the combination therapy reduced bilateral facial temperature difference (ΔT), ROI ΔT, and LBP histogram distance to a significantly greater extent than conventional acupuncture. The safety profiles of the two treatments were comparable, with no serious adverse events. These findings show an association between the combined intervention and more favorable short-term outcomes; they do not establish causality, component-specific efficacy, or safety equivalence.

Our results align with and extend previous research on acupuncture-based interventions for facial paralysis. Systematic reviews and meta-analyses have consistently shown that acupuncture improves clinical efficacy and shortens recovery time in acute and subacute peripheral facial paralysis (6, 11, 16). However, robust evidence specifically targeting the refractory population (duration ≥ 3 months, H-B grade ≥ III) has been sparse. Our study fills this gap by focusing exclusively on RPFP patients, a group characterized by extensive axonal degeneration, impaired nerve regeneration, and compromised facial microcirculation (5, 10). The observed superiority of precise meridian sinew needling over conventional acupuncture is consistent with the findings of a recent meta-analysis of 19 RCTs involving 1,436 patients, which reported that Jingjin (meridian sinew) acupuncture significantly improved overall effectiveness (OR = 3.93) and cure rate (RR = 1.69) compared with conventional acupuncture (29). Our results extend this literature by showing a favorable association for the combined treatment package in the present retrospective cohort. The three specific techniques we employed—Yangbai four-directional penetration, Dicang–Jiache palisade needling, and Taiyang to Dicang penetration—are designed to cover the motor endplate zones of frontal, periorbital, and perioral muscles, creating a three-dimensional stimulation network. This multi-target, multi-directional approach may be relevant to the observed differences, although the present study did not isolate individual treatment components or directly measure neural activation. The addition of warm acupuncture at Zusanli (ST36), Guanyuan (CV4), and Mingmen (GV4) is supported by clinical studies showing that warm acupuncture or thermosensitive moxibustion can improve total effective rates and reduce facial temperature asymmetry (24–26). A prospective trial of 84 elderly patients with refractory facial paralysis reported that warm acupuncture combined with low-frequency pulsed electrical stimulation significantly improved Sunnybrook scores and FDI subscales compared with warm acupuncture alone (24). Our study builds on these findings by using a more comprehensive protocol that combines warm acupuncture with meridian sinew needling, and by including objective ITI outcomes. Crucially, our study is one of the first to use infrared thermal imaging as a quantitative outcome measure in RPFP research. ITI has been increasingly recognized as a non-invasive, objective tool for assessing peripheral microcirculation and autonomic function (4, 31, 36). Previous studies have shown that facial temperature asymmetry correlates positively with the severity of facial paralysis, and that ITI parameters such as ROI ΔT and LBP histogram distance can discriminate between healthy individuals and patients with Bell’s palsy with high sensitivity and specificity (35, 36). Our finding that the combination therapy reduced bilateral ΔT from 0.49 °C to 0.26 °C—approaching the normal threshold of <0.2 °C—while the control group only reduced it to 0.32 °C, shows that the combination therapy was associated with lower facial surface-temperature asymmetry. This aligns with the TCM principle that “Qi and blood flow when warmed,” and is compatible with, but does not prove, the proposed theoretical rationale (19, 20).

Several findings in our study merit further discussion because they were not entirely anticipated. First, the magnitude of improvement in LBP histogram distance—a texture-based symmetry measure—was larger in the observation group (reduction from 0.69 to 0.39) than in the control group (0.66 to 0.48), with a between-group difference that was highly significant (*P* < 0.001). We had expected that bilateral ΔT and ROI ΔT, which directly reflect temperature differences, would improve, but the substantial improvement in LBP histogram distance was somewhat surprising. This parameter captures not just the mean temperature difference but the spatial distribution pattern of facial temperature, which may be more sensitive to subtle changes in microcirculation that are not fully captured by simple temperature differences. The LBP histogram distance has been used in facial paralysis assessment with promising results, but its application in evaluating treatment response is novel (35). Our data suggest that the combination therapy was associated with more symmetric surface-temperature patterns; blood flow, vasodilation, and collateral circulation were not measured. Second, the improvement in FDIS (social function) scores was larger in the observation group (from 61.02 to 85.22) than in the control group (60.17 to 77.93), with a between-group difference of 7.29 points (*P* < 0.001). While we expected better physical function (FDIP) in the observation group, the magnitude of the social function benefit was surprisingly large. The concurrent group-level improvements in FDIS and ITI parameters are clinically noteworthy but should be interpreted separately; without participant-level correlation analysis, this study cannot determine whether changes in thermal symmetry were associated with social or emotional gains. This finding is clinically important because social withdrawal and depression are common in patients with long-standing facial paralysis (2). The combination therapy, by achieving more complete facial functional recovery, may have a greater psychological impact than conventional acupuncture. Third, the safety data revealed that the observation group had a numerically but not statistically higher incidence of burns (2.41% vs. 1.20%) and infection (2.41% vs. 1.20%). Although the difference was not significant, the observation group involved moxibustion, which inherently carries a risk of thermal injury. We had anticipated that the burn rate might be higher, but the small number of events and wide confidence interval preclude a conclusion of equivalent or acceptable comparative safety. Nevertheless, this finding highlights the need for careful patient instruction and monitoring during warm acupuncture procedures.

Our findings have important implications for the theoretical understanding of refractory peripheral facial paralysis and its treatment. From a pathophysiological perspective, RPFP is characterized not only by persistent nerve conduction deficits but also by chronic microcirculatory impairment and neurogenic inflammation (9, 15). The facial nerve traverses a narrow bony canal (the fallopian canal), and any prolonged oedema or vasospasm within this confined space can lead to ischemic injury and subsequent fibrosis. Conventional acupuncture mainly targets nerve regeneration through local and distal point stimulation, but it may be insufficient to reverse the chronic ischemic changes that perpetuate the disease. Warm acupuncture adds a thermal component that can increase local blood flow, reduce blood viscosity, and promote the release of vasoactive substances such as calcitonin gene-related peptide (CGRP) and nitric oxide (NO), thereby improving the microcirculatory environment (23, 24). The greater improvement in ITI parameters is compatible with, but does not demonstrate, a vasodilatory mechanism because perfusion was not measured. The meridian sinew theory, as described in Ling Shu, posits that the sinews (jingjin) are the “walls” of the meridians and that diseases of the sinews manifest as either rigidity or flaccidity (21). In chronic facial paralysis, the affected side often presents with both flaccid (e.g., drooping mouth) and spastic (e.g., synkinesis) components. The precise meridian sinew needling techniques we used—especially the palisade needling along the orbicularis oris and buccinator muscles—are designed to “relax the sinews” by providing multi-point, low-intensity mechanical stimulation. This approach is analogous to the concept of “sensory input-driven motor reorganization” in neurorehabilitation, where widespread afferent stimulation enhances cortical plasticity and recruits surviving motor units (18, 28). From a neurophysiological perspective, the dense array of needles along the muscle fibers may activate intramuscular nerve endings, facilitating the release of neurotrophic factors such as brain-derived neurotrophic factor (BDNF) and glial cell line-derived neurotrophic factor (GDNF), which promote axonal sprouting and synaptic remodeling. Our clinical observation of improved Portmann scores (which include voluntary movements such as forehead wrinkling and mouth corner elevation) supports this neuroplasticity-based interpretation. Moreover, the combination of warm acupuncture at distal points (ST36, CV4, GV4) with local meridian sinew needling aligns with the TCM principle of “treating both the root and the branch.” The distal points, especially ST36, are known to regulate immune function and enhance systemic energy metabolism, while CV4 and GV4 are considered crucial for supplementing Qi and warming Yang. The significant improvement in TCM syndrome items such as sallow complexion, fatigue, and pale tongue in the observation group suggests that the systemic effects of warm acupuncture—perhaps mediated by the hypothalamic-pituitary-adrenal axis and the autonomic nervous system—contribute to the overall therapeutic outcome (19, 20). This integrated approach, which simultaneously addresses local nerve-muscle dysfunction and systemic Qi deficiency, may explain why the combination therapy outperformed conventional acupuncture, which focused primarily on local facial points and distal Hegu and Taichong. From a broader clinical perspective, our study demonstrates the value of incorporating objective biophysical measures such as infrared thermal imaging into TCM clinical research. One of the persistent criticisms of acupuncture research is the reliance on subjective outcome measures (e.g., patient-reported scales, clinician-graded scales) that are susceptible to bias. Although both ITI and clinical outcomes improved, participant-level correlations between changes in thermal metrics and changes in H-B grade, Portmann score, TCM syndrome scores, or FDI scores were not analyzed; parallel group-level changes therefore should not be interpreted as evidence of correlation. Future prospective studies should prespecify these correlation analyses to determine whether thermal changes track objective, clinician-rated, and patient-reported outcomes. This approach is consistent with the trend toward “integrative” and “precision” medicine, where multimodal assessment—including imaging, biomarkers, and patient-reported outcomes—is used to capture the full spectrum of treatment effects. Future studies could combine ITI with other neuroimaging modalities, such as functional near-infrared spectroscopy (fNIRS) or diffusion tensor imaging (DTI), to further elucidate the central and peripheral mechanisms of action. The proposed pathways involving nitric oxide, CGRP, BDNF, GDNF, neuroplasticity, immune regulation, and microcirculatory improvement were not measured; these explanations are hypotheses and should not be interpreted as mechanisms demonstrated by the present study.

Despite the strengths of this study—including the use of propensity score matching to reduce confounding, the inclusion of objective ITI outcomes, and a relatively large sample size (*n* = 166)—several limitations must be acknowledged. First and foremost, this was a retrospective study, not a prospective randomized controlled trial (RCT). Although propensity score matching balanced the baseline characteristics between groups, it cannot eliminate all unmeasured confounding factors (e.g., patients’ adherence to treatment, baseline psychological status, or subtle differences in acupuncture technique). The decision to use retrospective data was based on the practical challenges of recruiting RPFP patients, who are relatively rare, and the need to obtain real-world evidence. Nevertheless, the results should be confirmed in a well-designed, double-blind, sham-controlled RCT. We are currently planning such a trial. Second, the duration of follow-up was limited to the end of the 4-week treatment period. We did not assess long-term outcomes, such as relapse rates or sustained improvement at 3 or 6 months post-treatment. RPFP is a chronic condition, and it is possible that some of the gains observed immediately after treatment might diminish over time. Future studies should include longer follow-up periods to determine the durability of the treatment effect. Third, the blinding of patients and acupuncturists was not feasible given the nature of the interventions (patients in the observation group received additional moxibustion, which is easily distinguishable from conventional acupuncture). However, we did implement assessor blinding for all outcome evaluations, which reduces detection bias. Nevertheless, performance bias cannot be entirely ruled out. Fourth, the generalisability of our findings may be limited by the single-center design and the specific inclusion criteria (TCM pattern of Qi deficiency and blood stasis). Patients with other TCM patterns (e.g., wind-cold, wind-heat, or phlegm-stasis) might respond differently to the combination therapy. Moreover, the results may not be directly applicable to RPFP patients with comorbid conditions such as diabetes mellitus, hypertension, or autoimmune diseases, which were not specifically excluded but were not stratified. Multicentre studies with broader inclusion criteria are needed to validate the external validity of our findings. Fifth, the mechanistic interpretations we provided—involving vasodilation, neurotrophic factor release, and cortical plasticity—are speculative because we did not measure biomarkers or perform neurophysiological tests (e.g., electromyography, nerve conduction studies, or blood levels of BDNF/NO). In this study, ITI quantified facial surface-temperature distribution but could not establish improved microcirculation; direct perfusion and mechanistic measurements are needed to test the proposed pathways. Additional limitations include confounding by indication, residual post-matching imbalance in age and disease duration based on aggregate SMDs, lack of pre-matching balance diagnostics, incomplete reporting of etiology and comorbidities, use of analyses that did not account for matched pairs or ordinal outcomes, no multiplicity correction, inability to isolate treatment components, incomplete thermography calibration/reliability information, and uncertainty concerning the control-group burn entry. These issues restrict causal interpretation and require source-data verification where indicated.

Despite these limitations, our study provides preliminary observational evidence that warm acupuncture combined with precise meridian sinew needling may be a promising treatment for refractory peripheral facial paralysis. The integration of infrared thermal imaging offers an objective, quantifiable endpoint that could be used in future trials. We encourage researchers to replicate our findings in prospective, multicentre, sham-controlled RCTs with longer follow-up and mechanistic substudies. Such efforts are needed before this therapy can be considered a standard option for RPFP in both TCM and integrative medicine settings.

## 5 Conclusion

In this propensity score-matched retrospective study, warm acupuncture combined with precise meridian sinew needling was associated with more favorable 4-week clinical, functional, patient-reported, and facial thermal-symmetry outcomes than conventional acupuncture. The findings do not establish causality, the contribution of individual treatment components, increased blood flow, long-term effectiveness, or equivalent safety. Confirmation requires participant-level matched and baseline-adjusted analyses with effect estimates and 95% confidence intervals, followed by prospective multicentre studies with longer follow-up.

## Data Availability

The original contributions presented in this study are included in this article/supplementary material, further inquiries can be directed to the corresponding author.
